# Serum Levels of Human Neutrophil Peptides 1–3 (HNP1–3) as Potential Biomarkers in Psoriasis and Associated Comorbidities

**DOI:** 10.3390/biomedicines13071635

**Published:** 2025-07-03

**Authors:** Mateusz Mleczko, Anna Kowalska-Kępczyńska, Agnieszka Gerkowicz, Małgorzata Kowal, Dorota Krasowska

**Affiliations:** 1 Department of Dermatology, Venereology and Pediatric Dermatology, Medical University of Lublin, 20-059 Lublin, Poland; agnieszka.gerkowicz@umlub.pl (A.G.); malgorzata.kowal@umlub.pl (M.K.); dorota.krasowska@gmail.com (D.K.); 2 Department of Biochemical Diagnostics, Medical University of Lublin, 20-059 Lublin, Poland; anna.kowalska-kepczynska@umlub.pl

**Keywords:** psoriasis, human neutrophil peptides 1–3 (HNP1–3), systemic inflammation

## Abstract

**Background:** Psoriasis is a chronic inflammatory skin disease frequently associated with systemic comorbidities. Human neutrophil peptides 1–3 (HNP1–3), released by neutrophils, have both antimicrobial and proinflammatory effects and may contribute to the pathogenesis of psoriasis and its related conditions. The aim of this study was to evaluate the serum levels of HNP1–3 in patients with psoriasis compared with healthy controls and to assess their association with selected comorbidities and clinical parameters. **Methods:** In this cross-sectional study, forty-nine patients with psoriasis and forty-nine matched healthy controls were enrolled. Serum HNP1–3 levels were measured using ELISA. Clinical data, including waist-to-hip ratio (WHR), smoking status, and the presence of comorbidities such as psoriatic arthritis (PsA), cardiovascular disease, and pulmonary or autoimmune disorders, were recorded. **Results:** The mean HNP1–3 levels were significantly higher in the psoriasis patients than in the controls (3.85 ± 0.76 vs. 2.52 ± 0.84 ng/mL; *p* < 0.001), especially in patients with concomitant PsA (4.21 ± 0.69 ng/mL). Multivariable regression identified increased WHR (β = 1.77, *p* < 0.01) and smoking (β = 0.45, *p* < 0.001) as independent predictors of elevated HNP1–3 levels. Positive correlations were also found between HNP1–3 and ESR (r = 0.505, *p* = 0.019) and IL-6 (r = 0.561, *p* = 0.008). **Conclusions:** The elevated serum HNP1–3 levels identified in psoriasis patients—especially those with PsA, central obesity, and smoking history—suggest their potential utility as biomarkers of systemic inflammation. These findings highlight the systemic nature of psoriasis and warrant further research into the clinical utility of HNP1–3 in disease monitoring and risk stratification.

## 1. Introduction

Psoriasis is a chronic inflammatory skin disease that affects approximately 2–3% of the population [1]. It typically presents as symmetrically distributed, erythematous, thickened plaques covered with silvery scales [2]. Systemic inflammation in psoriasis has been linked to the development of various comorbid conditions [3]. Clinical observations and previous studies have shown that psoriasis is most frequently associated with psoriatic arthritis (PsA) [4] and, less commonly, cardiometabolic diseases [5,6,7,8,9,10], inflammatory bowel disease [11], pulmonary conditions [12,13,14,15,16,17,18,19], and psychological disorders such as depression [20]. These comorbidities significantly impair patients’ quality of life and contribute to the overall disease burden [21].

The pathogenesis of psoriasis—now considered an immune-mediated disorder—results from complex interactions between the innate and adaptive immune systems and is associated with the dysregulation of multiple inflammatory cytokines [22]. The importance of innate immunity is highlighted not only by epidermal barrier dysfunction, such as deletions of the late cornified envelope (LCE) genes [23], but also by the increased expression of antimicrobial peptides (AMPs) [24]. Previous studies have demonstrated elevated AMP expression and concentrations in psoriatic skin compared with healthy skin, with a particular emphasis on α-defensins [25,26,27].

**Figure 1:**
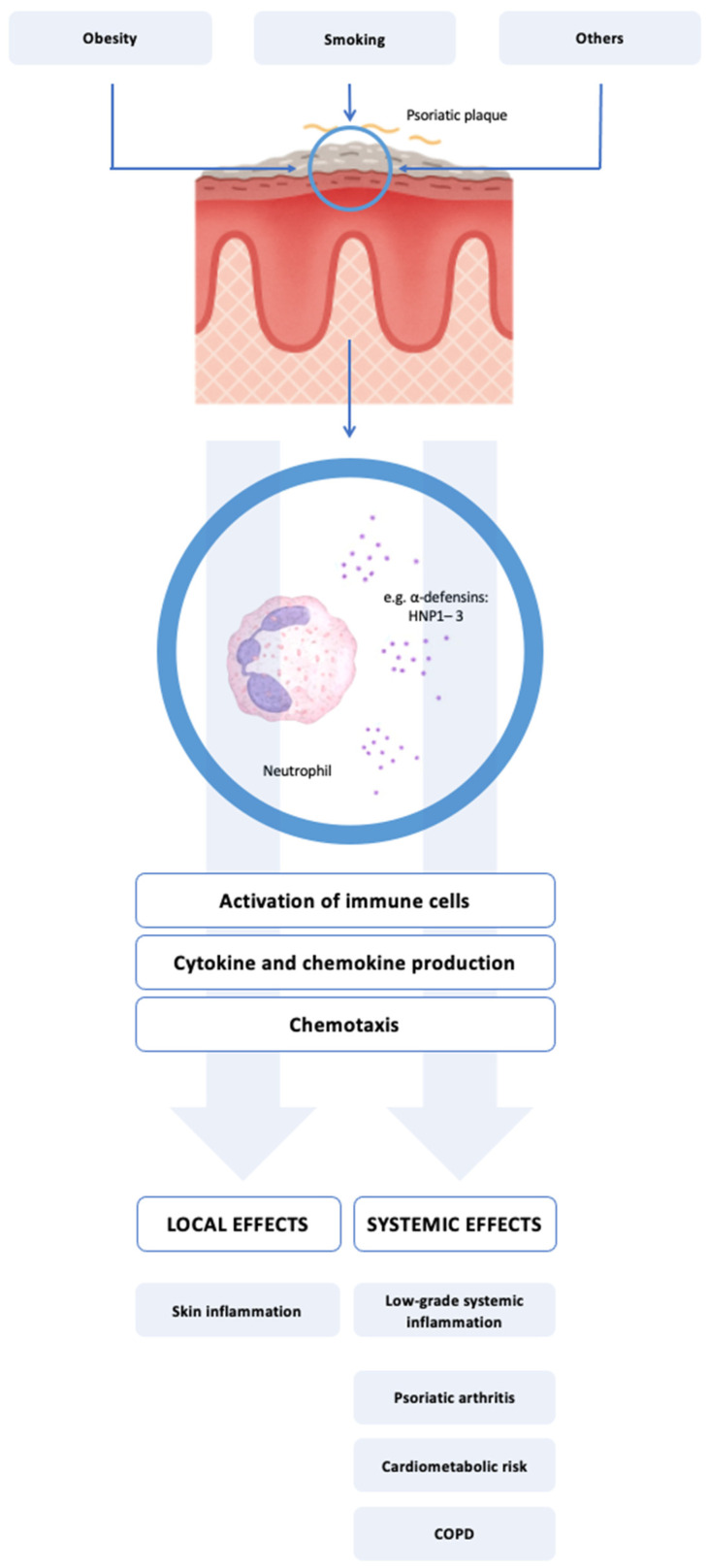
Proposed role of HNP1–3 in linking cutaneous and systemic inflammation in psoriasis. Neutrophils infiltrating psoriatic skin release HNP1–3, members of the α-defensin family with both antimicrobial and proinflammatory activity. These peptides contribute to local immune activation by stimulating dendritic cells, T cells, and keratinocytes, enhancing cytokine production and skin inflammation. A proportion of HNP1–3 enters systemic circulation, where it may contribute to low-grade systemic inflammation and be associated with comorbid conditions such as psoriatic arthritis, cardiovascular risk, and smoking-related lung disease (e.g., COPD).

α-Defensins, also known as human neutrophil peptides 1–3 (HNP1–3), are primarily stored in polymorphonuclear neutrophils (PMNs), and their release is markedly increased during acute, non-infectious inflammation [28]. This is consistent with the histopathological features of psoriasis, which include not only abnormal keratinocyte differentiation but also a dermal inflammatory infiltrate dominated by PMNs [29]. HNP1–3 are secreted by these cells and activate the innate immune response, potentially contributing to the pathogenesis of psoriasis and its associated comorbidities. Recent evidence suggests that HNP1–3 may influence Th17-mediated immune responses, potentially contributing to IL-17 axis activation. Although direct interactions between HNP1–3 and IL-17A have not been fully elucidated, these peptides are known to activate dendritic cells and promote the production of cytokines such as IL-6 and IL-23, which are crucial for differentiating and maintaining Th17 cells [30]. The potential role of HNP1–3 in linking cutaneous inflammation with systemic involvement is illustrated in Figure 1.

Although previous studies have documented increased HNP1–3 expression in psoriatic skin, data regarding their systemic concentrations and clinical significance in patients with psoriasis remain limited. Moreover, the potential association between circulating HNP1–3 levels and comorbid conditions in these patients is still unclear. This study advances the field by quantitatively analyzing serum HNP1–3 concentrations in a well-characterized cohort of patients with psoriasis, comparing them to matched healthy controls, and examining associations with key systemic inflammatory markers and comorbidities, including psoriatic arthritis, cardiovascular, and pulmonary diseases, as well as inflammation related to smoking and obesity.

Therefore, the aim of this study was to assess serum HNP1–3 levels in patients with psoriasis compared with healthy controls and to explore their association with selected comorbidities. To the best of our knowledge, this is the first study to examine serum HNP1–3 concentrations in relation to both psoriasis and its comorbidities. By focusing on the systemic role of neutrophil-derived peptides, this work expands the current understanding of psoriasis as a multi-organ inflammatory disease and identifies HNP1–3 as potential biomarkers for both cutaneous and extracutaneous disease burden. These findings offer novel insights that may aid future risk stratification and personalized disease monitoring strategies. However, given the observational design of this study, any identified associations between HNP1–3 and clinical variables should not be interpreted as causal. Further mechanistic and longitudinal research is needed to confirm these relationships.

## 2. Materials and Methods

### 2.1. Study Design

This cross-sectional, observational study was conducted between October 2020 and September 2023 at the Department of Dermatology, Venereology and Pediatric Dermatology, Medical University of Lublin, Poland. All patients underwent a thorough physical examination to identify any concurrent dermatological conditions. A detailed medical interview was performed, and a preliminary checklist was completed for each participant. All participants were informed about the study and provided written informed consent. Privacy and confidentiality were ensured by assigning each participant a unique identification code. The study protocol was approved by the Institutional Review Board and Ethics Committee of the Medical University of Lublin (approval no. KE-0254/294/2018). This study was conducted in accordance with the Declaration of Helsinki, Good Clinical Practice (GCP), and the International Conference on Harmonisation (ICH) guidelines.

### 2.2. Characteristics of the Study Population

The study group consisted of 49 patients over 18 years of age with clinically diagnosed psoriasis vulgaris, with or without concomitant PsA. The control group included 49 age- and sex-matched healthy individuals over 18 years of age. The inclusion criteria for the psoriasis group were as follows: confirmed diagnosis of psoriasis by a dermatologist, aged between 18 and 65 years, stable disease course (no recent flare-ups), and no recent initiation or change in systemic treatment. In uncertain cases, histopathological confirmation was used. The exclusion criteria for all participants included pregnancy or breastfeeding, active infection within the previous 3 months, use of systemic corticosteroids or immunosuppressive agents (unrelated to psoriasis) in the past 6 months, and a history of malignancy or chronic inflammatory diseases other than psoriasis.

### 2.3. Clinical Assessment

Participants underwent a comprehensive clinical examination, including medical history, assessment of the duration and severity of psoriasis, and evaluation for comorbidities. Psoriasis severity was assessed independently by two dermatologists using the Psoriasis Area and Severity Index (PASI); the average score was used for analysis. PsA was diagnosed according to the CASPAR classification criteria [31]. Demographic and clinical data were collected, including age, sex, disease history, concomitant diseases, smoking status, and current medications. Body mass index (BMI) was calculated according to the World Health Organization (WHO) criteria [32].

### 2.4. Blood Sample Collection and Biochemical Analyses

Fasting venous blood samples were collected from all participants to measure serum HNP1–3 concentrations. Blood was drawn into tubes containing a coagulation activator, allowed to clot at room temperature, and then centrifuged at 3500 rpm for 10 min at 25 °C. The resulting serum was aliquoted into polypropylene tubes and stored at −70 °C until analysis. Hemolyzed or lipemic samples were excluded.

Serum HNP1–3 concentrations were measured using a Human HNP1–3 (Neutrophil Peptide 1–3) ELISA Kit (Cat. No. EH3231; FineTest, Wuhan Fine Biotech Co., Ltd., Wuhan, China), based on the sandwich enzyme-linked immunosorbent assay (ELISA) principle. The assay was performed according to the manufacturer’s instructions. Briefly, standards and serum samples were added to wells pre-coated with a monoclonal antibody specific to HNP1–3. Following incubation and washing steps, a biotin-conjugated detection antibody and a horseradish peroxidase (HRP)–streptavidin solution were added. The colorimetric reaction was developed using a TMB substrate and stopped with an acidic stop solution. Absorbance was measured at 450 nm using the BioTek 800 TS Absorbance Reader. HNP1–3 concentrations were calculated based on a standard curve generated from the known concentrations provided in the kit. All measurements were performed in duplicate using Gen5 Microplate Reader and Imager (ver. 3.11, BioTek Instruments, Winooski, VT, USA).

In total, 7.6 mL of venous blood was also collected into a tube containing a coagulation activator, and biochemical parameters of inflammation were determined: ESR (erythrocyte sedimentation rate, [mm/h]), CRP (C-reactive protein, [mg/L]), and IL-6 (interleukin 6 [pg/mL]). The material was used within 2 h after collection. Biochemical parameters were determined using the COBAS 6000 biochemical analyzer (Roche Diagnostics, Warsaw, Poland).

### 2.5. Statistical Analysis

The data were analyzed using the Statistical Package for the Social Sciences (SPSS), version 29.0 (IBM Corp., Armonk, NY, USA). A formal a priori sample size calculation was not performed due to the exploratory and observational nature of this study. The number of participants was determined by the availability of eligible participants within the recruitment timeframe and logistical feasibility. Before conducting statistical analysis, the data were visually inspected to identify potential outliers. The normality of distribution was assessed using the Kolmogorov–Smirnov and Shapiro–Wilk tests. Continuous variables were compared using the independent *t*-test or Mann–Whitney U test, as appropriate. Categorical variables were analyzed using Pearson’s χ^2^ test. Correlations between HNP1–3 levels and clinical or laboratory parameters were evaluated using Pearson or Spearman correlation coefficients, depending on distribution. Multivariable linear regression was used to identify independent predictors of elevated HNP1–3 levels. Continuous data are presented as means ± standard deviations (SDs) and categorical data as frequencies and percentages. A *p*-value < 0.05 was considered statistically significant, and all confidence intervals were calculated at the 95% level.

## 3. Results

### 3.1. Baseline Characteristics of the Study Population

This study included 49 patients with psoriasis (mean age: 47.5 ± 12.6 years) and 49 age- and sex-matched healthy controls (mean age: 45.7 ± 10.9 years). There were no statistically significant differences between the groups regarding demographic, anthropometric, or clinical features. Detailed patient characteristics are presented in Table 1.

The mean duration of psoriasis was 17.6 ± 12.7 years. There was a positive family history of psoriasis in 18 (36.7%) cases and concomitant PsA in 6 (12.2%) cases. The mean PASI score was 15.8 ± 9.9. Previous medications included topical steroids/calcipotriol (n = 49 patients), ultraviolet B treatment (n = 12), biologics (n = 5), methotrexate (n = 13), cyclosporine (n = 12), acitretin (n = 6), and psoralen plus ultraviolet A (n = 4). The cumulative mean dose of methotrexate was 530 mg (from 120 to 1530 mg). The current medications were topical steroids/calcipotriol (n = 46 patients), ultraviolet B treatment (n = 2), and psoralen plus ultraviolet A (n = 0); no patients were receiving systemic therapy at the time of enrollment.

### 3.2. Serum HNP1–3 Levels and Selected Comorbid Conditions

Serum concentrations of HNP1–3 were significantly higher in patients with psoriasis than in healthy controls (3.85 ± 0.76 ng/mL vs. 2.52 ± 0.84 ng/mL; *p* < 0.001). Correlation analysis revealed no significant relationship between HNP1–3 levels and either disease duration or PASI score. Furthermore, among patients with psoriasis, those diagnosed with PsA exhibited the highest HNP1–3 concentrations (n = 12, 4.21 ± 0.69 ng/mL). Elevated serum HNP1–3 levels were also observed in individual psoriasis patients with rare or less common comorbidities, including coronary heart disease (n = 4), asthma (n = 2), COPD (n = 3), Crohn’s disease (n = 1), and systemic lupus erythematosus (n = 1). Serum HNP1–3 concentrations in healthy controls, patients with psoriasis, and subgroups with selected comorbid conditions are presented in Table 2.

### 3.3. Serum HNP1–3 Levels and Their Association with Clinical Parameters

Elevated serum HNP1–3 levels were also observed in psoriatic patients with specific clinical characteristics. Patients with an increased waist-to-hip ratio (WHR) and those with a history of smoking had significantly higher HNP1–3 concentrations than individuals without these features (3.97 ± 0.71 ng/mL and 3.90 ± 0.81 ng/mL, respectively; *p* < 0.001). In multivariable linear regression analysis, two independent predictors of elevated HNP1–3 levels were identified after adjusting for age and sex: increased WHR (β = 1.77, *p* < 0.01) and smoking status (β = 0.45, *p* < 0.001). The overall model was statistically significant (R^2^ = 0.64, *p* < 0.01) (Table 3).

### 3.4. Serum HNP1–3 Levels and Their Association with Biochemical Parameters

Moreover, associations were found between HNP1–3 parameters and the laboratory parameters of inflammation, including a significant positive correlation between serum HNP1–3 concentrations and ESR or IL-6 levels. The correlation coefficients were 0.505 for the ESR level (*p* = 0.019) and 0.561 for the IL-6 level (*p* = 0.008). The correlation of serum HNP1–3 with CRP, ESR, and IL-6 levels has been shown in Table 4. No other significant correlations between HNP1–3 and additional biochemical markers were observed.

## 4. Discussion

### 4.1. Clinical Relevance of Elevated HNP1–3 in Psoriasis and PsA

In this study, we found that the serum concentrations of human neutrophil peptides 1–3 (HNP1–3) were significantly elevated in patients with psoriasis compared with healthy controls. The highest levels were observed in patients with concomitant PsA, as well as in those with an increased WHR and smoking history. These findings support the hypothesis that HNP1–3 may be involved not only in the cutaneous immune response but also in systemic inflammation and psoriasis-associated comorbidities.

### 4.2. Associations with Clinical Features: WHR and Smoking

Elevated serum HNP1–3 levels were also observed in psoriatic patients with specific clinical characteristics. The association with central obesity may reflect a convergence of metabolic and immune pathways. Adipose tissue expresses neutrophil-attracting chemokines such as CXCL1 and CXCL2 and is itself a source of low-grade inflammatory mediators, including IL-6 and CRP [33]. Increased WHR correlates with elevated neutrophil activation, which may enhance HNP1–3 release into the circulation.

Cigarette smoking further amplifies neutrophil activity and defensin release by increasing oxidative stress and systemic inflammation [34]. Smoking has been linked to increased HNP1–3 levels in other inflammatory diseases [35]. Elevated HNP1–3 in smokers with psoriasis could thus reflect a synergistic effect for environmental and disease-related inflammatory stimuli. Moreover, beyond its general proinflammatory role, cigarette smoke has been shown to interfere with neutrophil maturation, apoptosis, and effector function [36]. Studies indicate that smoking delays neutrophil apoptosis and promotes the accumulation of aged, primed neutrophils with increased potential for degranulation and NET formation, processes directly associated with HNP1–3 release [37]. Additionally, cigarette smoke extract (CSE) promotes neutrophil survival through PI3K/Akt-mediated signaling and enhances the release of granular proteins, including α-defensins [38]. These effects may contribute to elevated HNP1–3 levels and persistent low-grade systemic inflammation observed in smokers with psoriasis.

### 4.3. Correlations with Inflammatory Biomarkers

While classical biomarkers such as CRP and IL-6 are widely used in psoriasis-related research and clinical monitoring, each has limitations. CRP primarily reflects acute-phase hepatic responses and is not consistently elevated in all psoriasis patients, particularly in the absence of systemic flares. IL-6 plays a central role in stimulating hepatic CRP production, yet it also participates more broadly in systemic immune modulation, including neutrophil recruitment and activation [39]. Therefore, the direct association between HNP1–3 and IL-6 may reflect shared upstream inflammatory pathways, particularly those involving neutrophil-driven cytokine cascades [40]. However, IL-6 is not routinely measured in clinical practice due to technical and cost limitations, despite its therapeutic relevance.

By contrast, HNP1–3 are neutrophil-derived peptides with both antimicrobial and immunomodulatory functions that may reflect neutrophil activation and low-grade systemic inflammation. In our study, HNP1–3 correlated with IL-6 and ESR but not with CRP, suggesting that they may capture a distinct aspect of inflammatory activity, possibly linked to chronic innate immune activation. Therefore, HNP1–3 could serve as complementary biomarkers alongside existing inflammatory markers, particularly in patients with comorbidities or features of systemic involvement not adequately captured by CRP alone. Further studies are needed to determine whether HNP1–3 can enhance clinical decision-making or predict therapeutic responses.

### 4.4. Mechanistic Implications: HNP1–3 as Immunomodulatory Mediators

Our findings align with the known biology of HNP1–3, which are α-defensins secreted predominantly by polymorphonuclear neutrophils (PMNs) during degranulation or NETosis. They may also be expressed at lower levels by other cell types, such as monocytes, certain epithelial cells, and natural killer (NK) cells, under inflammatory conditions [41,42]. However, neutrophils remain their primary source in the circulation. In addition to their well-established antimicrobial activity, these peptides exert potent immunomodulatory functions. They stimulate the release of proinflammatory cytokines such as TNF-α, IL-1β, and IL-8 from monocytes and macrophages [43], enhance the maturation of dendritic cells [44], and promote the chemotaxis of T cells via interaction with CCR6 receptors [45]. These mechanisms contribute to amplifying both innate and adaptive immune responses characteristic of psoriatic inflammation.

HNP1–3 can also directly affect keratinocyte biology. In vitro studies have shown that these peptides induce keratinocyte proliferation and the production of cytokines such as IL-6 and IL-8 [46], which are key mediators of the psoriatic cytokine milieu. By promoting IL-6 release, HNP1–3 may indirectly influence Th17 cell polarization and IL-17-mediated inflammatory cascades, which are central to psoriatic pathology [47]. This potential connection warrants further investigation, especially in the context of biologic therapies targeting the IL-17/IL-23 axis. This creates a positive feedback loop of neutrophil recruitment, peptide release, and epidermal activation, perpetuating chronic inflammation in the skin. Importantly, HNP1–3 can form complexes with self-DNA and RNA released during cell damage. These complexes activate Toll-like receptors (TLR9 and TLR7/8) in plasmacytoid dendritic cells, triggering the production of type I interferons [48], which are implicated in early psoriatic lesion formation and may also contribute to systemic autoimmunity. Moreover, TLR activation, particularly in myeloid and dendritic cells, is known to enhance IL-6 and IL-23 production, both of which are essential for Th17 cell differentiation and IL-17A secretion [49]. While a direct link between HNP1–3 and IL-17 production has not yet been firmly established, these mechanistic intermediates support the hypothesis that HNP1–3 contributes to IL-17-driven inflammation via TLR-dependent innate immune activation.

### 4.5. Relevance in Psoriatic Arthritis and Other Comorbidities

The elevated HNP1–3 levels observed in patients with PsA are consistent with prior reports suggesting a role for neutrophil-derived peptides in joint inflammation, such as myeloperoxidase and calprotectin [27]. Neutrophils are present in the synovial fluid of PsA patients, and experimental data suggest they may release extracellular traps (NETs) containing HNP1–3, which could contribute to the formation of autoantigens and activation of synovial fibroblasts [50,51]. However, HNP1–3′s involvement in these mechanisms remains speculative and was not directly evaluated in our study. Elevated serum concentrations of HNP1–3 have also been observed in other systemic inflammatory disorders, supporting their broader role as circulating biomarkers of immune activation.

In rheumatoid arthritis (RA), increased serum HNP1–3 levels correlate with disease activity and joint destruction, likely reflecting enhanced neutrophil degranulation and NET formation within synovial tissues [50]. Similarly, studies of patients with inflammatory bowel disease (IBD), particularly Crohn’s disease, have reported elevated plasma HNP1–3 levels, which may reflect both mucosal neutrophil infiltration and systemic inflammatory spillover [52]. In systemic lupus erythematosus (SLE), serum HNP1–3 have been implicated in triggering type I interferon responses via Toll-like receptor pathways, contributing to systemic autoimmunity [48]. These findings suggest that serum HNP1–3 is a standard marker of innate immune activation across multiple chronic inflammatory conditions. The elevated levels in patients in our study with comorbidities such as coronary heart disease, asthma, COPD, Crohn’s disease, and systemic lupus erythematosus further support this hypothesis. However, given the limited number of cases in these subgroups, the results should be interpreted with caution.

### 4.6. Study Limitations

Owing to its cross-sectional and observational design, our study cannot establish causal relationships. While the identified associations between HNP1–3 levels and clinical parameters such as PsA, central obesity, and smoking are intriguing, they must be interpreted with caution. Longitudinal studies are essential to determine whether elevated HNP1–3 is a consequence or a potential contributor to systemic inflammation in psoriasis.

Several limitations should be noted. First, the sample size was relatively small, particularly in subgroup analyses (e.g., asthma, Crohn’s disease, and systemic lupus erythematosus), limiting the statistical power of subgroup analyses and precluding definitive conclusions for these conditions. Second, owing to the cross-sectional design, we could not assess longitudinal changes in HNP1–3 levels or their predictive value. Third, while we observed associations with selected comorbidities, further studies with larger cohorts and mechanistic approaches are needed to confirm causality. Finally, while ELISA is a sensitive method, variability in assay kits and lack of standardized reference ranges may affect reproducibility across studies and contribute to potential sources of bias.

Furthermore, our cross-sectional design could not determine whether elevated HNP1–3 levels precede the clinical onset of psoriasis. While our findings demonstrate elevated serum concentrations in patients with established disease, it remains unknown whether HNP1–3 can serve as early indicators of subclinical inflammation or predictors of disease development in genetically susceptible individuals. Future prospective longitudinal studies are needed to assess whether HNP1–3 elevation occurs prior to the appearance of clinical symptoms and could be used as a predictive biomarker in at-risk populations.

## 5. Conclusions

This study demonstrated that serum concentrations of human neutrophil peptides 1–3 (HNP1–3) are significantly elevated in patients with psoriasis compared with healthy controls, with the highest levels observed in those with PsA. Increased HNP1–3 levels were also associated with specific concomitant conditions, particularly central obesity and smoking. These findings support the concept of psoriasis as a systemic inflammatory disease rather than a purely cutaneous condition. The serum concentrations of HNP1–3 could aid in the early identification of patients at increased risk of comorbidities and guide more comprehensive clinical assessment and personalized management.

Further longitudinal studies are needed to validate the clinical utility of HNP1–3 and explore their role in disease monitoring, risk stratification, and therapeutic response in psoriasis and related systemic conditions. Moreover, the potential role of HNP1–3 as early biomarkers preceding disease onset warrants further investigation in longitudinal, at-risk cohorts. Significantly, the observational nature of this study limits causal inference, and the role of HNP1–3 as biomarkers versus mechanistic mediators remains to be elucidated.

## Figures and Tables

**Table 1:** Demographic, clinical, and biochemical features of the study participants. PASI—Psoriasis Area Severity Index; PsA—psoriatic arthritis; BMI—body mass index; WHR—waist-to-hip ratio; AHT—arterial hypertension; DM—diabetes mellitus; CHD—coronary heart disease; COPD—chronic obstructive pulmonary disease; SLE—systemic lupus erythematous; CRP—C-reactive protein; ESR—erythrocyte sedimentation rate; IL-6—interleukin-6; ns—non-significant; N/A—not applicable.

Feature	Psoriasis Patients (n = 49)	Controls (n = 49)	*p*-Value
**Demographic**			
Age, years ± SD	47.5 ± 12.6	45.7 ± 10.9	ns
Male, n (%)	36 (73.5)	38 (77.6)	ns
**Clinical**			
Psoriasis duration, years ± SD	17.6 ± 12.7	N/A	
PASI, mean ± SD	15.8 ± 9.9	N/A	
PsA, n (%)	12 (24.5)	N/A	
WHR, mean ± SD	0.96 ± 0.1	0.93 ± 0.1	ns
BMI, mean ± SD (kg/m^2^)	28.3 ± 4.7	27.4 ± 2.9	ns
Hyperlipidemia, n (%)	21 (42.8)	24 (49)	ns
AHT, n (%)	15 (30.1)	17 (34.7)	ns
DM, n (%)	4 (8.1)	6 (12.2)	ns
CHD, n (%)	4 (8.1)	2 (4)	ns
COPD, n (%)	3 (6.1)	4 (8.1)	ns
Asthma, n (%)	2 (4)	2 (4)	ns
Crohn’s disease, n (%)	1 (2)	0 (0)	ns
SLE, n (%)	1 (2)	0 (0)	ns
Smoking cigarettes, n (%)	18 (36.7)	15 (30.6)	ns
Cigarette pack-years, mean ± SD	16.9 ± 13	14.1 ± 10.1	ns
Intensity (cigarettes/day), mean ± SD	14.8 ± 7	13.2 ± 8.8	ns
Respiratory infections in last 6 weeks, n (%)	2 (4)	2 (4)	ns
**Biochemical**			
ESR, mean ± SD (mm/h)	26.15 ± 19.46	21.09 ± 20.8	ns
CRP, mean ± SD (mg/L)	6.62 ± 7.86	5.01 ± 5.81	ns
IL-6, mean ± SD (pg/mL)	4.86 ± 10.31	3.12 ± 11.02	ns

**Table 2:** Serum concentrations of HNP1–3 in healthy controls, patients with psoriasis, and selected comorbid subgroups. Data for rare comorbidities are shown as individual observations. AHT—arterial hypertension; PsA—psoriatic arthritis; CHD—coronary heart disease; DM—diabetes mellitus; COPD—chronic obstructive pulmonary disease; SLE—systemic lupus erythematous; ns—non-significant; N/A—not applicable. * Individual values exceed the control group mean and, in some cases, the mean value for the overall psoriasis cohort.

Group	n	Mean HNP1–3 Concentration ± SD (ng/mL)	*p* Values
Healthy controls	49	2.52 ± 0.84	
Psoriasis (total)	49	3.85 ± 0.76	<0.001
with AHT	15	3.49 ± 0.41	ns
with PsA	12	4.21 ± 0.69	<0.001
with CHD	4	3.89 ± 0.81	N/A *
with DM	4	3.71 ± 0.91	N/A
with COPD	3	4.12 ± 0.71	N/A *
with asthma	2	4.05 ± 0.55	N/A *
with Crohn’s disease	1	4.01	N/A *
with SLE	1	4.14	N/A *

**Table 3:** Multivariable linear regression analysis of factors associated with elevated serum HNP1–3 levels in patients with psoriasis. WHR—waist-to-hip ratio; ns—non-significant.

Variable	β Coefficient	95% CI	*p*-Value
WHR	1.77	0.62–2.91	<0.01
Smoking status	0.45	0.21–0.69	<0.001
Age (adjusted)	-	-	ns
Sex (adjusted)	-	-	ns

**Table 4:** Correlation of serum HNP1–3 with CRP, ESR, and IL-6 levels. CRP—C-reactive protein; ESR—erythrocyte sedimentation rate; IL-6—interleukin-6.

Variable Pair	Pearson r	*p*-Value
HNP1–3 vs. CRP	−0.089	0.701
HNP1–3 vs. ESR	0.505	0.019
HNP1–3 vs. IL-6	0.561	0.008

## Data Availability

The original contributions presented in this study are included in the article. Further inquiries can be directed to the corresponding author(s).

## References

[1] Boehncke W.H., Schön M.P. (2015). Psoriasis. Lancet.

[2] Rendon A., Schäkel K. (2019). Psoriasis Pathogenesis and Treatment. Int. J. Mol. Sci..

[3] Daudén E., Castañeda S., Suárez C., García-Campayo J., Blasco A.J., Aguilar M.D., Ferrándiz C., Puig L., Sánchez-Carazo J.L. (2013). Clinical Practice Guideline for an Integrated Approach to Comorbidity in Patients with Psoriasis. J. Eur. Acad. Dermatol. Venereol..

[4] Liu J.-T., Yeh H.-M., Liu S.-Y., Chen K.-T. (2014). Psoriatic Arthritis: Epidemiology, Diagnosis, and Treatment. World J. Orthop..

[5] Coto-Segura P., Eiris-Salvado N., González-Lara L., Queiro-Silva R., Martinez-Camblor P., Maldonado-Seral C., García-García B., Palacios-García L., Gomez-Bernal S., Santos-Juanes J. (2013). Psoriasis, Psoriatic Arthritis and Type 2 Diabetes Mellitus: A Systematic Review and Meta-Analysis. Br. J. Dermatol..

[6] Armstrong A.W., Harskamp C.T., Armstrong E.J. (2013). The Association between Psoriasis and Hypertension: A Systematic Review and Meta-Analysis of Observational Studies. J. Hypertens..

[7] Armstrong A.W., Harskamp C.T., Armstrong E.J. (2012). The Association between Psoriasis and Obesity: A Systematic Review and Meta-Analysis of Observational Studies. Nutr. Diabetes.

[8] Miller I.M., Ellervik C., Yazdanyar S., Jemec G.B.E. (2013). Meta-Analysis of Psoriasis, Cardiovascular Disease, and Associated Risk Factors. J. Am. Acad. Dermatol..

[9] Gu W.J., Weng C.L., Zhao Y.T., Liu Q.H., Yin R.X. (2013). Psoriasis and Risk of Cardiovascular Disease: A Meta-Analysis of Cohort Studies. Int. J. Cardiol..

[10] Van Der Voort E.A.M., Koehler E.M., Dowlatshahi E.A., Hofman A., Stricker B.H., Janssen H.L.A., Schouten J.N.L., Nijsten T. (2014). Psoriasis Is Independently Associated with Nonalcoholic Fatty Liver Disease in Patients 55 Years Old or Older: Results from a Population-Based Study. J. Am. Acad. Dermatol..

[11] Fu Y., Lee C.-H., Chi C.-C. (2018). Association of Psoriasis With Inflammatory Bowel Disease: A Systematic Review and Meta-Analysis. JAMA Dermatol..

[12] Wang J., Ke R., Shi W., Yan X., Wang Q., Zhang Q., Chai L., Li M. (2018). Association between Psoriasis and Asthma Risk: A Meta-Analysis. Allergy Asthma Proc..

[13] Ungprasert P., Srivali N., Thongprayoon C. (2016). Association between Psoriasis and Chronic Obstructive Pulmonary Disease: A Systematic Review and Meta-Analysis. J. Dermatol. Treat..

[14] Li X., Kong L., Li F., Chen C., Xu R., Wang H., Peng S., Zhou M., Li B. (2015). Association between Psoriasis and Chronic Obstructive Pulmonary Disease: A Systematic Review and Meta-Analysis. PLoS ONE.

[15] Ger T.-Y., Fu Y., Chi C.-C. (2020). Bidirectional Association Between Psoriasis and Obstructive Sleep Apnea: A Systematic Review and Meta-Analysis. Sci. Rep..

[16] Choi Y.M., Famenini S., Wu J.J. (2017). Incidence of Pulmonary Arterial Hypertension in Patients with Psoriasis: A Retrospective Cohort Study. Perm. J..

[17] Mleczko M., Gerkowicz A., Krasowska D. (2024). Co-Occurrence of Psoriasis and Asthma in the Pediatric Population: A Systematic Review and Meta-Analysis. J. Clin. Med..

[18] Ishikawa G., Dua S., Mathur A., Acquah S.O., Salvatore M., Beasley M.B., Padilla M.L. (2019). Concomitant Interstitial Lung Disease with Psoriasis. Can. Respir. J..

[19] Khalid U., Gislason G.H., Hansen P.R. (2014). Sarcoidosis in Patients with Psoriasis: A Population-Based Cohort Study. PLoS ONE.

[20] Lukmanji A., Basmadjian R.B., Vallerand I.A., Patten S.B., Tang K.L. (2021). Risk of Depression in Patients With Psoriatic Disease: A Systematic Review and Meta-Analysis. J. Cutan. Med. Surg..

[21] Bu J., Ding R., Zhou L., Chen X., Shen E. (2022). Epidemiology of Psoriasis and Comorbid Diseases: A Narrative Review. Front. Immunol..

[22] Lowes M.A., Suárez-Fariñas M., Krueger J.G. (2014). Immunology of Psoriasis. Annu. Rev. Immunol..

[23] de Cid R., Riveira-Munoz E., Zeeuwen P.L.J.M., Robarge J., Liao W., Dannhauser E.N., Giardina E., Stuart P.E., Nair R., Helms C. (2009). Deletion of the Late Cornified Envelope LCE3B and LCE3C Genes as a Susceptibility Factor for Psoriasis. Nat. Genet..

[24] Hilchie A.L., Wuerth K., Hancock R.E.W. (2013). Immune Modulation by Multifaceted Cationic Host Defense (Antimicrobial) Peptides. Nat. Chem. Biol..

[25] Harder J., Dressel S., Wittersheim M., Cordes J., Meyer-Hoffert U., Mrowietz U., Fölster-Holst R., Proksch E., Schröder J.-M., Schwarz T. (2010). Enhanced Expression and Secretion of Antimicrobial Peptides in Atopic Dermatitis and after Superficial Skin Injury. J. Investig. Dermatol..

[26] Wittmann M., McGonagle D., Werfel T. (2014). Cytokines as Therapeutic Targets in Skin Inflammation. Cytokine Growth Factor Rev..

[27] Bierkarre H., Harder J., Cuthbert R., Emery P., Leuschner I., Mrowietz U., Hedderich J., McGonagle D., Gläser R. (2016). Differential Expression of Antimicrobial Peptides in Psoriasis and Psoriatic Arthritis as a Novel Contributory Mechanism for Skin and Joint Disease Heterogeneity. Scand. J. Rheumatol..

[28] Yang D., Biragyn A., Hoover D.M., Lubkowski J., Oppenheim J.J. (2004). Multiple Roles of Antimicrobial Defensins, Cathelicidins, and Eosinophil-Derived Neurotoxin in Host Defense. Annu. Rev. Immunol..

[29] Ogawa E., Sato Y., Minagawa A., Okuyama R. (2018). Pathogenesis of Psoriasis and Development of Treatment. J. Dermatol..

[30] Rycyk-Bojarzyńska A., Kasztelan-Szczerbińska B., Cichoż-Lach H., Surdacka A., Roliński J. (2024). Human Neutrophil Alpha-Defensins Promote NETosis and Liver Injury in Alcohol-Related Liver Cirrhosis: Potential Therapeutic Agents. J. Clin. Med..

[31] Taylor W., Gladman D., Helliwell P., Marchesoni A., Mease P., Mielants H. (2006). Classification Criteria for Psoriatic Arthritis: Development of New Criteria from a Large International Study. Arthritis Rheum..

[32] (1995). Physical Status: The Use and Interpretation of Anthropometry, Report of a WHO Expert Committee.

[33] Wensveen F.M., Valentić S., Šestan M., Turk Wensveen T., Polić B. (2015). The “Big Bang” in Obese Fat: Events Initiating Obesity-Induced Adipose Tissue Inflammation. Eur. J. Immunol..

[34] Qiu S.-L., Zhang H., Tang Q.-Y., Bai J., He Z.-Y., Zhang J.-Q., Li M.-H., Deng J.-M., Liu G.-N., Zhong X.-N. (2017). Neutrophil Extracellular Traps Induced by Cigarette Smoke Activate Plasmacytoid Dendritic Cells. Thorax.

[35] Wang Z., Yao W.-Z., Xia G.-G., Sun D.-J. (2008). The expression and implications of human alpha-defensin 1-3 in serum and induced sputum in patients with chronic obstructive pulmonary disease. Zhonghua Jie He He Hu Xi Za Zhi Chin. J. Tuberc. Respir. Dis..

[36] Heijink I.H., Pouwels S.D., Leijendekker C., de Bruin H.G., Zijlstra G.J., van der Vaart H., ten Hacken N.H.T., van Oosterhout A.J.M., Nawijn M.C., van der Toorn M. (2015). Cigarette Smoke-Induced Damage-Associated Molecular Pattern Release from Necrotic Neutrophils Triggers Proinflammatory Mediator Release. Am. J. Respir. Cell Mol. Biol..

[37] Quinn M.T., Gauss K.A. (2004). Structure and Regulation of the Neutrophil Respiratory Burst Oxidase: Comparison with Nonphagocyte Oxidases. J. Leukoc. Biol..

[38] Hosseinzadeh A., Thompson P.R., Segal B.H., Urban C.F. (2016). Nicotine Induces Neutrophil Extracellular Traps. J. Leukoc. Biol..

[39] Gabay C., Kushner I. (1999). Acute-Phase Proteins and Other Systemic Responses to Inflammation. N. Engl. J. Med..

[40] Németh T., Mócsai A. (2012). The Role of Neutrophils in Autoimmune Diseases. Immunol. Lett..

[41] Huang S.U.-S., O’Sullivan K.M. (2022). The Expanding Role of Extracellular Traps in Inflammation and Autoimmunity: The New Players in Casting Dark Webs. Int. J. Mol. Sci..

[42] Fang Q., Stehr A.M., Naschberger E., Knopf J., Herrmann M., Stürzl M. (2022). No NETs No TIME: Crosstalk between Neutrophil Extracellular Traps and the Tumor Immune Microenvironment. Front. Immunol..

[43] Yang D., Chertov O., Bykovskaia S.N., Chen Q., Buffo M.J., Shogan J., Anderson M., Schröder J.M., Wang J.M., Howard O.M. (1999). Beta-Defensins: Linking Innate and Adaptive Immunity through Dendritic and T Cell CCR6. Science.

[44] Funderburg N., Lederman M.M., Feng Z., Drage M.G., Jadlowsky J., Harding C.V., Weinberg A., Sieg S.F. (2007). Human β-Defensin-3 Activates Professional Antigen-Presenting Cells via Toll-like Receptors 1 and 2. Proc. Natl. Acad. Sci. USA.

[45] Rodríguez-Frade J.M., del Real G., Serrano A., Hernanz-Falcón P., Soriano S.F., Vila-Coro A.J., de Ana A.M., Lucas P., Prieto I., Martínez-A C. (2004). Blocking HIV-1 Infection via CCR5 and CXCR4 Receptors by Acting in Trans on the CCR2 Chemokine Receptor. EMBO J..

[46] Ong P.Y., Ohtake T., Brandt C., Strickland I., Boguniewicz M., Ganz T., Gallo R.L., Leung D.Y.M. (2002). Endogenous Antimicrobial Peptides and Skin Infections in Atopic Dermatitis. N. Engl. J. Med..

[47] Takahashi T., Yamasaki K. (2020). Psoriasis and Antimicrobial Peptides. Int. J. Mol. Sci..

[48] Lande R., Ganguly D., Facchinetti V., Frasca L., Conrad C., Gregorio J., Meller S., Chamilos G., Sebasigari R., Riccieri V. (2011). Neutrophils Activate Plasmacytoid Dendritic Cells by Releasing Self-DNA-Peptide Complexes in Systemic Lupus Erythematosus. Sci. Transl. Med..

[49] Gaffen S.L., Jain R., Garg A.V., Cua D.J. (2014). IL-23-IL-17 Immune Axis: Discovery, Mechanistic Understanding, and Clinical Testing. Nat. Rev. Immunol..

[50] Curran A.M., Naik P., Giles J.T., Darrah E. (2020). PAD Enzymes in Rheumatoid Arthritis: Pathogenic Effectors and Autoimmune Targets. Nat. Rev. Rheumatol..

[51] Papadaki G., Kambas K., Choulaki C., Vlachou K., Drakos E., Bertsias G., Ritis K., Boumpas D.T., Thompson P.R., Verginis P. (2016). Neutrophil Extracellular Traps Exacerbate Th1-Mediated Autoimmune Responses in Rheumatoid Arthritis by Promoting DC Maturation. Eur. J. Immunol..

[52] Wehkamp J., Salzman N.H., Porter E., Nuding S., Weichenthal M., Petras R.E., Shen B., Schaeffeler E., Schwab M., Linzmeier R. (2005). Reduced Paneth Cell Alpha-Defensins in Ileal Crohn’s Disease. Proc. Natl. Acad. Sci. USA.

